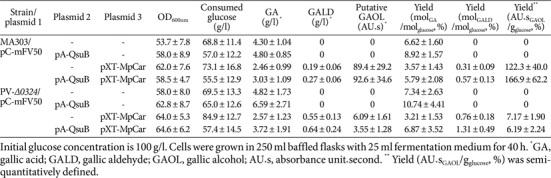# Metabolic Engineering of *Corynebacterium glutamicum* for the Fermentative Production of Gallic Compounds by Extending the Shikimate Pathway

**DOI:** 10.4014/jmb.2409.04009

**Published:** 2025-06-12

**Authors:** Min-Hee Jung, Jung-Min Choi, Theavita Chatarina Mariyes, Eun-Jae Ju, Jin-Ho Lee

**Affiliations:** Department of Food Science and Biotechnology, BB21+, Food and Life Science Research Institute, Kyungsung University, Busan 48434, Republic of Korea

**Keywords:** Gallic acid, gallic aldehyde, gallic alcohol, *Corynebacterium glutamicum*, metabolic engineering

## Abstract

Gallic acid, gallic aldehyde, and gallic alcohol are polyphenolic compounds with promising antioxidant and therapeutic properties. Despite their biological significance, a complete microbial biosynthetic route for their production from simple carbon sources has not been established. We engineered *Corynebacterium glutamicum* to produce gallic acid and its two reduced derivatives *via* a synthetic pathway extended from the shikimate pathway. Introduction of a mutant 4-hydroxybenzoate hydroxylase conferred protocatechuate hydroxylation activity in *C. glutamicum*. Among tested mutants, the Y385F/L200V mutant exhibited the highest gallic acid production, reaching 4.03 g/l with a yield of 5.95% in flask cultures. To enable gallic aldehyde biosynthesis, carboxylic acid reductases (CARs) from various microbial sources were screened. Of these, MpCAR exhibited the highest catalytic activity toward gallic acid, producing 0.66 g/l of gallic aldehyde in an *NCgl0324*-deleted strain. Further reduction of gallic aldehyde to gallic alcohol was achieved using the endogenous aromatic aldehyde reductase encoded by *NCgl0324* in *C. glutamicum*, as confirmed by Q-TOF mass analysis. Overexpression of *qsuB* encoding 3-dehydroshikimate dehydratase improved carbon flux from 3-dehydroshikimate toward PCA and significantly enhanced the gallic compound production. In 5-l fed-batch fermentation, engineered strains produced up to 12.0 g/l gallic acid, 1.14 g/l gallic aldehyde, and 172.4 AU*s gallic alcohol, respectively, representing 82-86% increases compared to flask cultures. This study reports the first complete microbial biosynthetic route for gallic acid, gallic aldehyde, and gallic alcohol from D-glucose. Our work highlights *C. glutamicum* as a robust microbial platform for sustainable production of value-added gallic polyphenols through pathway design and metabolic engineering.

## Introduction

Gallic acid (3,4,5-trihydroxybenzoic acid) is a naturally occurring polyphenol widely found in plants such as gallnuts, sumac, witch hazel, tea leaves, and oak bark [1, 2]. It exhibits diverse biological activities, including antioxidants, antibacterial, antiviral, anti-allergic, and anti-inflammatory effects [1, 3, 4]. These properties have led to its applications in the pharmaceutical industry for therapeutic agent development and in the food industry as a natural preservative [2, 4, 5]. Gallic aldehyde (3,4,5-trihydroxybenzaldehyde), a derivative of gallic acid found in *Geum japonicum*, has been reported to exhibit antioxidant activity and to inhibit MMP-2 and MMP-9 expression as well as the migration of human aortic smooth muscle cells [6, 7]. These effects suggest its potential as a bioactive compound for vascular disease prevention, cancer suppression, and oxidative stress reduction. Additionally, gallic aldehyde has been shown to protect astrocytes from oxygen-glucose deprivation/reperfusion-induced injury by reducing astrocyte reactivity and apoptosis [8]. Gallic alcohol (3,4,5-trihydroxybenzyl alcohol) is another derivative of gallic acid, but its biosynthetic pathway has not been reported. While its biological functions remain largely unexplored, its structural similarity to 4-hydroxybenzyl (4-HB) alcohol, a compound known for its antioxidant and neuroprotective effects, suggests that gallic alcohol may possess various bioactive properties [9, 10].

Conventional methods for gallic acid production rely on the hydrolysis of tannin-rich plant materials using acids or bases [11]. Additionally, microbial whole-cell catalytic systems have been used to convert tannin or lignin into gallic acid [11, 12]. However, these methods are constrained by factors such as limited raw material availability, harsh reaction conditions, environmental issues, and/or low conversion efficiency [13, 14]. Despite these existing methods, a natural microbial biosynthetic pathway for gallic acid production from glucose has not been established, primarily due to the absence of a hydroxylase capable of catalyzing the conversion of protocatechuate (PCA, 3,4-dihydroxybenzoate) to gallic acid. Previous studies demonstrated that substituting tyrosine-385 with phenylalanine in 4-hydroxybenzoate hydroxylase (PobA) from *Pseudomonas aeruginosa* enabled PCA conversion to gallic acid [15]. Moreover, additional mutations introduced alongside Y385F, such as T294A, T294A/V349A, and L199V, further enhanced hydroxylation activity toward PCA [14, 16, 17]. More recently, Guo *et al*. established an artificial biosynthetic pathway for gallic acid production from 3-dehydroshikimate (3-DHS) by introducing QuiC (3-dehydroshikimate dehydratase) along with the mutant PobA (Y385F/T294A/V349A) [18]. To further enhance gallic acid production, metabolic engineering strategies were implemented to increase the availability of phosphoenolpyruvate and erythrose 4-phosphate as well as to boost 3-DHS supply. The engineered *Escherichia coli* strain achieved a high gallic acid titer, producing 51.6 g/l in a 3-l fermenter with a carbon yield of 0.45 g/g. While significant progress has been made in engineering microorganisms for gallic acid production, efforts to extend this approach to the biosynthesis of its reduced derivatives, gallic aldehyde and gallic alcohol, remain unexplored. To date, no naturally occurring microorganisms have been reported to reduce gallic acid to gallic aldehyde and gallic alcohol, nor have studies been conducted on engineering microbial strains for their production.

**Fig. 1:**
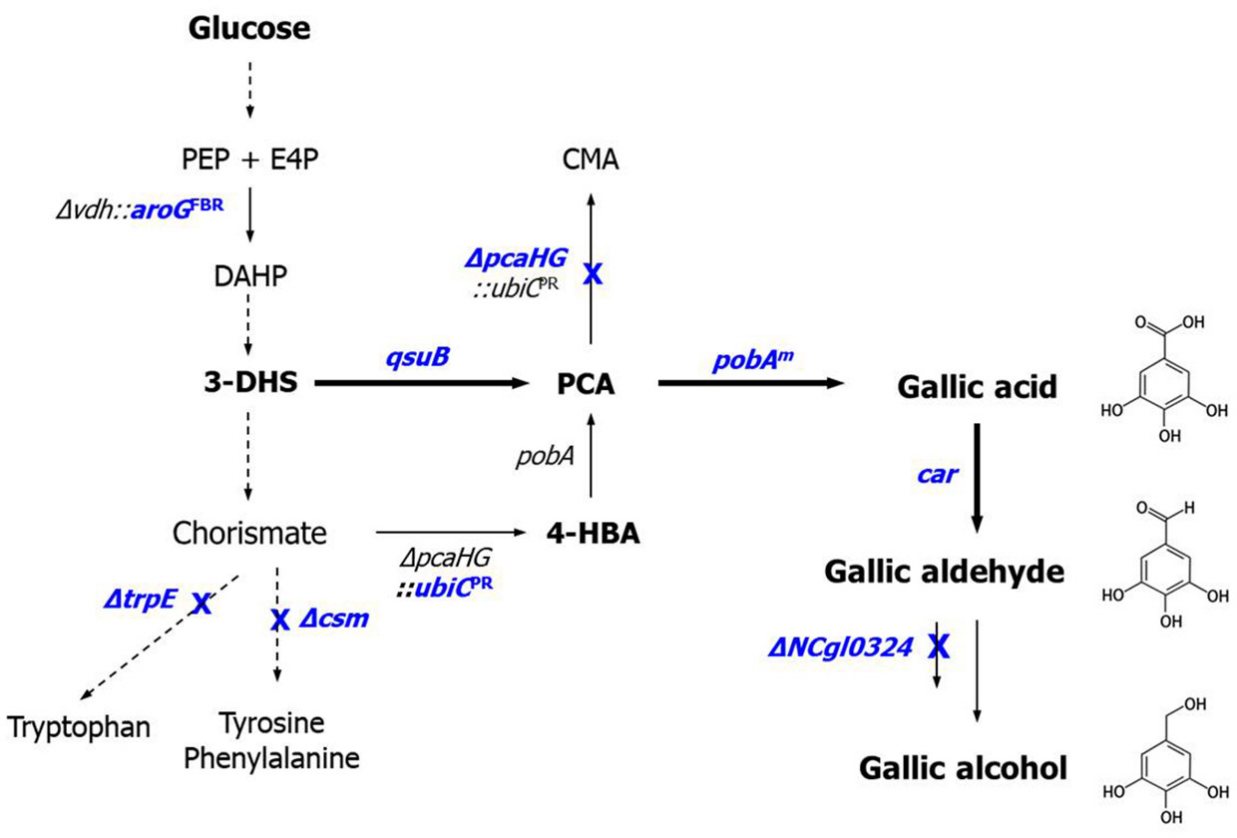
Schematic representation of metabolically engineered *C. glutamicum* producing gallic compounds. Bold arrows represent catalytic steps by plasmid-borne expression of corresponding genes; crosses indicate the disruption of corresponding genes. Dashed arrows show several reaction steps. The abbreviations are as follows: PEP, phosphoenolpyruvate; E4P, erythrose 4-phosphate; DAHP, 3-deoxy-D-arabinoheptulosonate-7-phosphate; 3-DHS, 3-dehydroshikimate; 4-HBA, 4- hydroxybenzoate; PCA, protocatechuate; CMA, β-carboxymuconate. Genes and correspoding enzymes are as follows: *aroG*^FBR^,a feedback-resistant DAHP synthase from *E. coli*; *trpE*, anthranilate synthase; *csm*, chorismate mutase; *pcaHG*, protocatechuate dioxygenase; *ubiC*^PR^, a product-resistant chorismate-pyruvate lyase from *E. coli*; *pobA*, 4-hydroxybenzoate hydroxylase; *pobA*^m^, a mutant 4-hydroxybenzoate hydroxylase; car, carboxylic acid reductase ; *NCgl0324*, aromatic aldehyde reductase.

*Corynebacterium glutamicum* is a widely utilized industrial microbial chassis for the production of amino acids, organic acids, alcohols, and amines [19-21]. Beyond these applications, *C. glutamicum* has gained attention as a promising host for synthesizing shikimate pathway-derived aromatic acids, including 4-hydroxybenzoate (4-HBA), PCA, and vanillic acid [22-26]. Building on this foundation, the introduction of carboxylic acid reductase (CAR) has facilitated the biosynthesis of corresponding aromatic alcohols, such as 4-HB alcohol, protocatechuic (PC) alcohol, and vanillyl alcohol. More recently, *C. glutamicum* was engineered to accumulate aromatic aldehydes, including 4-hydroxybenzaldehyde (4-HB aldehyde), PC aldehyde, and vanillin, by expressing the *car* gene while mitigating their conversion into alcohols through the deletion of *NCgl0324* encoding aromatic aldehyde reductase (AAR) [26]. These advancements underscore the potential of *C. glutamicum* as a versatile synthetic biology platform for producing a wide range of aromatic acids, aldehydes, and alcohols by extending the shikimate pathway and fine-tuning CAR and AAR activities. This study represents the first attempt to establish a complete biosynthetic pathway from PCA to gallic aldehyde and gallic alcohol in *C. glutamicum* by extending the chorismate-derived pathway through the introduction of CAR and modulation of AAR (Fig. 1). Additionally, enhancing metabolic rerouting *via* *qsuB* overexpression was employed to improve the conversion of 3-DHS into PCA, thereby increasing the biosynthetic efficiency of gallic compounds. These findings highlight the potential of *C. glutamicum* as an industrially relevant microbial host for the sustainable production of gallic acid and its derivatives, expanding its applicability in the microbial production of value-added polyphenols.

## Materials and Methods

### Bacterial Strains, Plasmids, and General Genetic Techniques

The bacterial strains and plasmids utilized in this study are summarized in Tables 1 and 2. *E. coli* Top10 and BL21(DE3) were employed as host strains for vector construction and overexpression of the *pobA* gene, respectively. *C. glutamicum* MA303 and PV-*Δ0324* served as parent strains for the production of gallic acid, its precursors, and derivatives [26]. Plasmids pCXE50T, pCES208, and pALT601 were used for gene expression in *C. glutamicum*, while pET-24a(+) was utilized for *pobA* expression and purification in *E. coli*. DNA fragments were amplified by polymerase chain reaction (PCR) using TOPsimple DryMIX-Tenuto (Enzynomics, Republic of Korea). For the assembly of DNA fragments, an EZ-fusion HT Cloning Kit (Enzynomics) was employed based on the Gibson assembly method [27]. PCR-amplified fragments incorporated into vectors were verified through sequencing. *C. glutamicum* transformation was conducted *via* electroporation [28].

### Media and Culture Conditions

Fermentation for the production of gallic acid, its precursors, and derivatives was carried out in 250-ml baffled flasks using the previously reported flask fermentation medium and conditions [23, 26], with the modification that 100 g/l of D-glucose was used as the carbon source. When necessary, the culture medium was supplemented with 50 mg/l kanamycin, 4.8 mg/l chloramphenicol, and/or 50 mg/l spectinomycin. To prevent the oxidative degradation of gallic compounds after glucose depletion, the cultivation was terminated at 40 h under conditions where residual glucose remained. Cell growth was monitored by measuring the optical density at 600 nm (OD_600_) using a UV-2550 spectrophotometer (Shimadzu, Japan). All experiments were performed in triplicate, and the mean values along with standard deviations were reported. For 5-l bioreactor fermentation, the medium composition and operational conditions were based on previously reported protocols [23]. However, to prevent the oxidation of gallic acid, glucose feeding was conducted before complete depletion of the initially supplied D-glucose, when its concentration dropped below 20 g/l.

### Plasmid Construction for Mutant 4-Hydroxybenzoate Hydroxylase from *C. glutamicum*

Primer lists used in this work are summarized in Table S1. To express *pobA* from *C. glutamicum*, the open reading frame (ORF) of *pobA* (1.188 kb) was amplified by PCR using the primer set P-F1/P-R2 and genomic DNA from *C. glutamicum* ATCC13032 as a template. The purified DNA fragment was subsequently cloned into pCXE50T at the *Eco*RI/*Hin*dIII sites, generating pXT-wPO (Table 2). For the construction of plasmids carrying single amino acid substitutions in PobA, site-directed mutagenesis was employed. The Y385F mutation (Tyr385→Phe; TAC → TTC) was introduced by amplifying two overlapping fragments using the primer sets P-F3/P-R4 and P-F5/P-R6. The resulting amplicons were assembled into pXT-wPO digested with *Pst*I and *Nhe*I, yielding pXT-mF385. Similarly, the T294A mutation (Tyr294→Ala; ACC → GCC) was introduced by amplifying two fragments using the primer sets P-F3/P-R8 and P-F7/P-R6, followed by cloning into the same vector, resulting in pXT-mA294. The L200V mutation (Lys200→Val; CTC → GTG) was generated using PCR fragments amplified with the primer sets P-F1/P-R10 and P-F9/P-R2, which were subsequently assembled into pCXE50T at the *Eco*RI/*Hin*dIII sites, producing pXT-mV200. For the construction of plasmids carrying double-mutant variants of PobA, three DNA fragments were assembled into recombinant plasmids. The Y385F/L200V double mutation was generated by amplifying using the primer sets P-F1/P-R10, P-F9/P-R4, and P-F5/P-R6, which were subsequently cloned into pCXE50T/*Eco*RI/*Hin*dIII, generating pXT-mFV50. Likewise, the Y385F/T294A double mutation was constructed by amplifying using the primer sets P-F3/P-R8, P-F7/P-R4, and P-F5/P-R6, followed by cloning into pCXE50T/*Pst*I/*Nhe*I sites, producing pXT-mFA54. To introduce PobA variants into a plasmid carrying a kanamycin resistance marker, all five recombinant plasmids were digested with *Not*I and *Kpn*I, releasing 1.7 kb DNA fragments containing the *tuf* promoter, *pobA* ORF, and *rrnB* terminator. These fragments were then ligated into pCES208 linearized with the same restriction enzymes, generating pC-wPO, pC-mF385, pC-mV200, pC-mA294, pC-mFV50, and pC-mFA54. To purify His-tagged PobA enzymes, the previously constructed vectors pXT-wPO, pXT-mF385, pXT-mV200, and pXT-mFV50 were used as template DNA. The *pobA* genes were amplified *via* PCR using the primer set P-F11/P-R12, and the resulting amplicons were cloned into the pET24a(+)/ vector digested with *Nde*I and *Xho*I *via* Gibson assembly. The final constructs were designated as pET-wPO, pET-mF385, pET-mV200, and pET-mFV50, respectively.

### Plasmid Construction for Expression of car and *qsuB* Genes

Codon-optimized versions of the *car* genes from *Mycobacterium phlei* (*MpCar*, 3.489 kb), *Neurospora crassa* OR74A (*NcCar*, 3.159 kb), and *Segniliparus rotundus* DSM 44985 (*SrCar*, 3.561 kb) were synthesized by Integrated DNA Technologies (IDT, USA) and cloned into pCXE50T at the *Eco*RI/*Hin*dIII sites (Tables S2-S4). The resulting plasmids were designated as pXT-MpCar, pXT-NcCar, and pXT-SrCar, respectively (Table 2). Additionally, the *qsuB* gene (1.857 kb) from *C. glutamicum* was amplified using the primer set P-F13/P-R14 and cloned into pCXE50T at the *Eco*RI/*Hin*dIII sites, generating pXT-QsuB. The *qsuB* region, including its promoter and terminator from pXT-QsuB, was then subcloned into pCES208, resulting in pA-QsuB.

### Purification and Enzyme Assay of 4-Hydroxybenzoate Hydroxylase Mutants

*E. coli* BL21(DE3) harboring pET-wPO, pET-mF385, pET-mV200, or pET-mFV50 was cultivated in 300 ml of LB medium supplemented with kanamycin at 37°C with shaking at 200 rpm. Protein expression was induced with 1 mM IPTG at OD_600_ = 0.5, followed by incubation at 18°C for 12 h. Cells were harvested by centrifugation, washed with PBS, and resuspended in buffer W (20 mM Tris-HCl, 400 mM NaCl, pH 7.4). Cell disruption was performed by sonication on ice, and the lysate was centrifuged to remove debris. The supernatant containing the soluble fraction was applied to a Ni-NTA affinity chromatography column pre-equilibrated with buffer W. After 1 h of incubation to allow for His-tagged protein binding, unbound proteins were removed by washing with buffer W. Additional washing with buffer E (20 mM Tris-HCl, 300 mM NaCl, 500 mM imidazole) was conducted to remove proteins bound non-specifically. Target proteins were eluted using a stepwise imidazole gradient by increasing the buffer E to buffer W ratio sequentially: 99:1, 98:2, 95:5, 90:10, and finally 50:50. The final elution step (50:50) was repeated four times, yielding fractions E1 to E4. The presence and integrity of the purified proteins were confirmed by SDS-PAGE analysis (data not shown).

The hydroxylation activity of the His-tagged PobA variants was assessed using two approaches: one based on NADPH oxidation and the other on product formation (PCA or gallic acid). For both assays, the reaction mixture contained 50 mM Tris-HCl (pH 8.0), 0.2 mM EDTA, 2 mM dithiothreitol, 2 μM FAD, 0.2 mM NADPH, and 5 mM of the substrate (either 4-HBA or PCA). The final volume was adjusted to 1 mL with enzyme and water. NADPH oxidation was monitored spectrophotometrically at 340 nm to track NADPH consumption, and the enzyme activity was calculated as μmol NADPH oxidized per min (unit, U) per mg protein, using an extinction coefficient of 6.22 × 10³/M/cm. In the second approach, product formation was quantified by determining the conversion of 4-HBA or PCA into PCA or gallic acid over time. Aliquots (200 μl) were collected at three predefined time points, and the reaction was immediately quenched with 0.1 N HCl to prevent further enzymatic activity. Samples were diluted (1:5) in methanol and subjected to high-performance liquid chromatography (HPLC) analysis. Product formation activity was defined as the amount of enzyme required to produce 1 μmol of PCA or gallic acid per min (U) per mg protein. All experiments were performed in triplicate.

### Analytical Procedures

For HPLC quantification of the fermentation broth, the samples were prepared by diluting them 100-fold using methanol as the solvent. The diluted samples were centrifuged, and only the supernatant was filtered before injecting 10 μl into the HPLC system. Analysis was performed using an Agilent 1260 system. Separation was carried out using an Eclipse XDB-C18 column (4.6 × 250 mm, 5 μm), which contains a non-polar stationary phase. A gradient method was applied with a polar mobile phase consisting of 0.1% phosphoric acid in deionized water and 0.1% phosphoric acid in acetonitrile. The flow rate was maintained at 0.8 ml/min, and the column oven temperature was set to 25°C. The initial mobile phase composition (0 min) was 95:5 (0.1% phosphoric acid in deionized water:0.1% phosphoric acid in acetonitrile), which was maintained for 20 min. Over the next 30 min, the solvent composition was gradually changed to 15:85, followed by an immediate shift to 0:100. This condition was maintained for 2 min, after which the system was programmed to return to the initial solvent composition over 3 min. Using a DAD detector, 4-HBA, 4-HB aldehyde, 4-HB alcohol, PCA, PC aldehyde, PC alcohol, gallic acid, and gallic aldehyde were detected at 280 nm, while putative gallic alcohol was detected at 230 nm. The mass analysis of putative gallic alcohol was performed using LC-QTOF-MS in negative ion mode, following the sample preparation and analytical protocols described by Putri *et al*. [29].

## Results

### Production of Gallic Acid by Introduction of Mutant 4-Hydroxybenzoate Hydroxylase from *C. glutamicum*

The wild-type PobA catalyzes the hydroxylation of 4-HBA at the C3 position, resulting in the production of PCA. However, the wild-type enzyme exhibits no activity for further hydroxylation of PCA at the C5 position to generate gallic acid [17]. Previous studies on PobA from *P. aeruginosa* have demonstrated that specific mutations, such as Y385F, Y385F/T294A, and Y385F/L199V, confer enzymatic activity capable of converting PCA to gallic acid [14, 16, 17]. Based on this knowledge, we aimed to engineer *C. glutamicum* for gallic acid production by introducing mutant forms of PobA from *C. glutamicum*, including Y385F, L200V, T294A, Y385F/L200V, and Y385F/T294A. To achieve this, *C. glutamicum* strains were constructed by cloning mutated *pobA* genes into the pCX50T vector and subsequently subcloning them into the pCES208 vector. The resulting strains were cultivated in baffled flasks containing 100 g/l of D-glucose, and the production of gallic acid and its precursors was evaluated after 40 h (Table 3). The parental strain, *C. glutamicum* MA303, which lacks any engineered PobA, produced negligible amounts of gallic acid. The MA303 harboring pC-wPO (expressing wild-type *pobA*) produced approximately 0.56 g/l of gallic acid, indicating minimal intrinsic activity of the wild-type enzyme to convert PCA to gallic acid. Among strains harboring single-mutant variants of PobA, the T294A and L200V mutants led to diminished gallic acid accumulation (0.28 and 0.26 g/l, respectively) compared to strains harboring the pC-wPO plasmid. Notably, the Y385F mutant significantly enhanced gallic acid production, yielding 3.88 g/l (yield 5.39%), while also reducing PCA accumulation to 0.54 g/l. Similar to the Y385F variant from *P. aeruginosa* [15], this result highlights the crucial role of the Y385F mutation in *C. glutamicum* PobA in altering substrate specificity, shifting PobA activity toward PCA hydroxylation into gallic acid. Strains synthesizing double-mutant PobA variants displayed varied results. Unlike the Y385F/T294A mutant from *P. aeruginosa*, MA303 harboring the Y385F/T294A mutant from *C. glutamicum* produced 3.75 g/l of gallic acid (yield 4.87%), suggesting that the T294A mutation did not positively impact the PCA hydroxylation activity of Y385F. In contrast, the Y385F/L200V double mutant led to the highest gallic acid production, with a titer of 4.03 g/l (yield 5.95%), indicating a synergistic effect between Y385F and L200V in enhancing PCA hydroxylation activity. These results demonstrate that the Y385F mutation is a key determinant in conferring PCA hydroxylation activity to PobA from *C. glutamicum*. While the L200V mutation alone contributes minimally to this activity, its combination with Y385F enhances enzyme efficiency, resulting in the highest gallic acid yield observed in this study. In contrast, the T294A mutation did not positively affect PCA hydroxylation, particularly when combined with Y385F.

### Hydroxylation Activity of 4-Hydroxybenzoate Hydroxylase Mutants

To further investigate the role of PobA mutations in facilitating gallic acid production, the corresponding mutant proteins (Y385F, L200V, and Y385F/L200V) were purified, and their hydroxylation activity toward 4-HBA and PCA was assessed (Tables 4 and 5). Wild-type PobA exhibited strong hydroxylation activity toward 4-HBA, as evidenced by high NADPH oxidation (2.765 U/mg protein) and PCA production (1.742 U/mg protein). However, when PCA was used as a substrate, wild-type PobA showed no detectable gallic acid production despite significant NADPH oxidation (5.401 U/mg protein), confirming that PCA hydroxylation does not occur. These results indicate that wild-type PobA from *C. glutamicum*, similar to previously characterized PobA enzymes from *Pseudomonas* species, is highly specific for 4-HBA hydroxylation and does not catalyze further hydroxylation of PCA to gallic acid [14]. Compared to wild-type PobA, the Y385F mutant exhibited significantly reduced 4-HBA hydroxylation activity, as indicated by lower NADPH oxidation (0.324 U/mg protein) and decreased PCA production (0.206 U/mg protein). Additionally, NADPH oxidation was lower for both 4-HBA (0.322 U/mg protein) and PCA (0.681 U/mg protein), consistent with an overall decrease in redox activity. However, the Y385F mutation conferred PCA hydroxylation activity, enabling low-efficiency gallic acid production at 0.183 U/mg protein. In contrast, the L200V mutant resulted in approximately a 6-fold increase in NADPH oxidation (15.87 U/mg protein) and a high PCA production rate (5.444 U/mg protein). However, it did not exhibit PCA hydroxylation activity, indicating that while this mutation enhances the first hydroxylation step (4-HBA to PCA), it does not contribute to the second hydroxylation step (PCA to gallic acid). The double mutant Y385F/L200V exhibited 58%and 97% increases in NADPH oxidation for 4-HBA (0.512 U/mg protein) and PCA (1.344 U/mg protein), respectively, compared to Y385F. In addition, Y385F/L200V led to a substantial increase in gallic acid formation activity (0.642 U/mg protein), exhibiting a 251% increase compared to Y385F. Overall, these results demonstrate that the Y385F mutation is crucial for conferring PCA hydroxylation activity and shifting PobA specificity from 4-HBA to PCA. Moreover, the combination of Y385F and L200V effectively redirects PobA activity away from 4-HBA hydroxylation and toward PCA hydroxylation, significantly improving gallic acid production.

### Biosynthesis of Gallic Aldehyde by Introduction of Carboxylic Acid Reductase

To extend the metabolic pathway beyond gallic acid, a carboxylic acid reductase (CAR) capable of reducing gallic acid to gallic aldehyde was introduced (Fig. 1). Since CARs exhibit broad substrate specificity depending on their microbial origin [30, 31], we aimed to identify the most effective enzyme by expressing different *car* genes in *C. glutamicum*. To achieve this, *car* genes from *Mycobacterium phlei* (*MpCar*), *Neurospora crassa* (*NcCar*), *Segniliparus rotundus* (*SrCar*), and *Nocardia iowensis* (*NiCar*) were selected from the literature [32-35]. Codon-optimized versions of *MpCar*, *NcCar*, and *SrCar* were cloned into pCXE50T, generating the expression plasmids pXT-MpCar, pXT-NcCar, and pXT-SrCar, respectively (Table 2). Additionally, the previously constructed pICA4335, renamed pXI-*NiCar*, was included in this analysis. To evaluate the catalytic efficiency of these CARs in reducing gallic acid, the constructed plasmids were introduced into *C. glutamicum* PV-*Δ0324*, a strain in which *NCgl0324* was deleted to minimize aromatic aldehyde reduction, thereby facilitating gallic aldehyde accumulation while suppressing gallic alcohol formation. The engineered strains, co-harboring the plasmid pC-mFV50 for gallic acid biosynthesis, were cultivated in baffled flasks containing 100 g/l of D-glucose, and their ability to convert gallic acid into gallic aldehyde was evaluated (Table 6). Among the tested CARs, the *MpCar*-expressing GC300 strain exhibited the highest gallic aldehyde accumulation, reaching 0.66 g/l (yield 0.96%), followed by *NiCar*, which accumulated 0.14 g/l (yield 0.22%). In contrast, strains expressing *NcCar* or *SrCar* did not produce detectable levels of gallic aldehyde, indicating that these enzymes have little or no catalytic activity toward gallic acid. The gallic acid consumption patterns further supported this trend, as strains expressing *MpCar* or *NiCar* exhibited higher substrate utilization, while strains expressing *NcCar* or *SrCar* showed minimal gallic acid consumption, confirming their inefficiency in catalyzing the reduction of gallic acid to gallic aldehyde. These findings establish a functional artificial biosynthetic pathway for gallic aldehyde production from PCA in *C. glutamicum* PV-*Δ0324* by introducing the PobA mutant (Y385F/L200V) and MpCAR.

### Biosynthesis of Putative Gallic Alcohol *via* Endogenous Aromatic Aldehyde Reductase

**Fig. 2:**
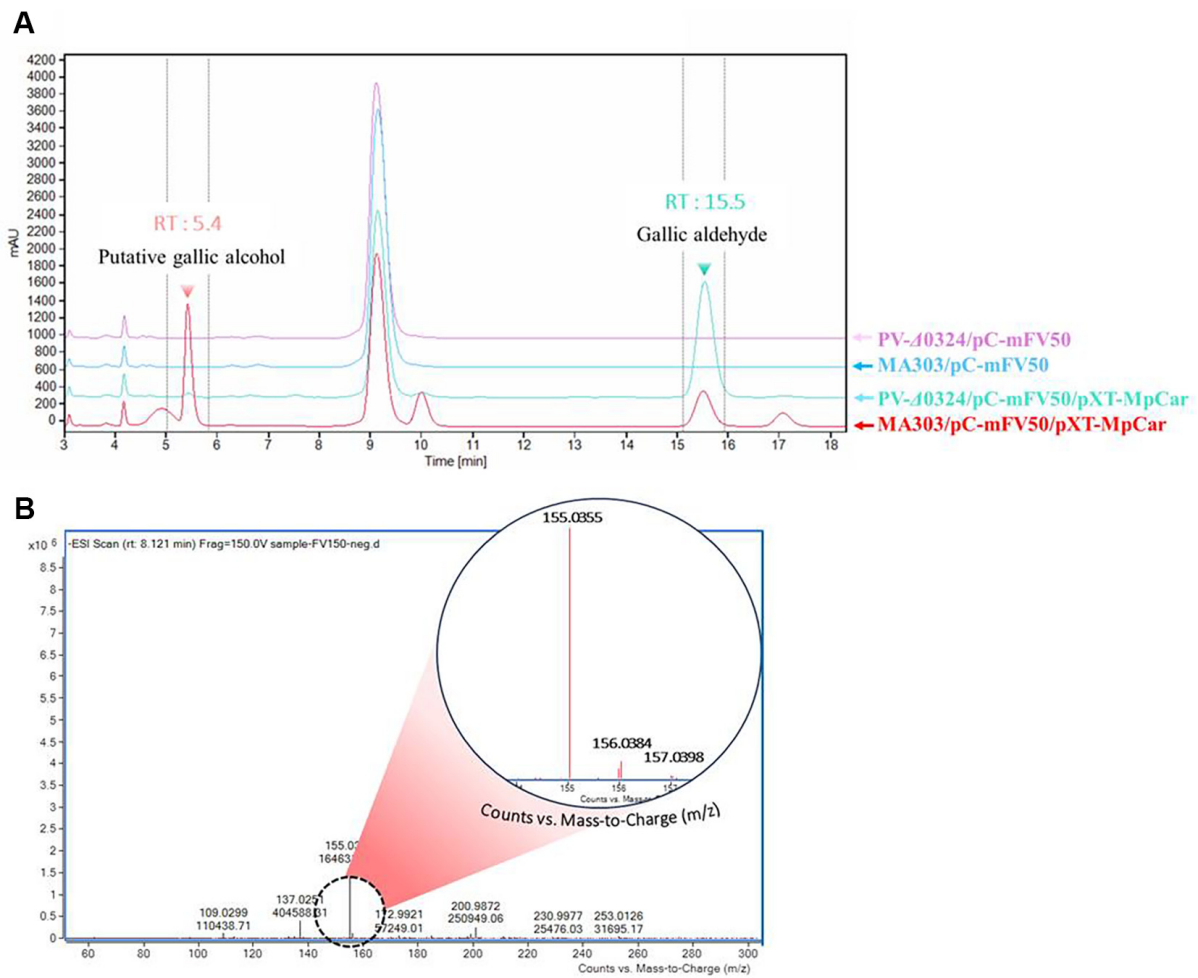
Comparative analysis of HPLC chromatograms (A) and mass analysis of putative gallic alcohol by LCQTOF- MS (B).

Gallic aldehyde synthesized *via* the pathway established in this study is expected to be further reduced to gallic alcohol by endogenous aromatic aldehyde reductase(s) in *C. glutamicum* (Fig. 1) [26]. To evaluate this conversion, MpCAR and NiCAR, which exhibited high efficiency in reducing gallic acid, were introduced into *C. glutamicum* PC150 (MA303/pC-mFV50), a strain retaining the *NCgl0324* gene. The engineered strains were cultivated in baffled flasks containing 100 g/l of D-glucose, and their ability to convert gallic acid into gallic aldehyde and subsequently into gallic alcohol was evaluated (Table 7). Since gallic alcohol is not commercially available, direct quantification was not feasible. However, a comparative analysis of HPLC chromatograms of two engineered strains - GC307 (*NCgl0324*-deleted PV-*Δ0324* with pC-mFV50 and pXT-MpCar) and GC152 (*NCgl0324*-retaining MA303 with pC-mFV50 and pXT-MpCar) - revealed distinct differences (Fig. 2A). In strain GC152, the gallic aldehyde peak (retention time: 15.5 min) was significantly reduced compared to GC307, while a pronounced unknown peak with a retention time of 5.4 min was observed. To identify this unknown compound, the corresponding HPLC fraction was collected and analyzed using Q-TOF mass spectrometry. In negative ion mode, two peaks were detected at [M-H]^-^ = 155.0355 and 156.0384, which are consistent with the theoretical monoisotopic mass of gallic alcohol (156.0422 Da) (Fig. 2B). These findings strongly suggest that the highly increased peak at RT 5.4 min corresponds to putative gallic alcohol. Among the tested strains, the *MpCar*-expressing GC152 strain exhibited the highest gallic alcohol production (85.8 AU*s), followed by the *NiCar*-expressing strain (21.2 AU*s)(Table 6). These results indicate that MpCAR efficiently reduces gallic acid to gallic aldehyde, which is subsequently converted to gallic alcohol by endogenous aromatic aldehyde reductase(s). The successful biosynthetic conversion of gallic acid to gallic alcohol in *C. glutamicum* GC152 demonstrates a functional and efficient two-step enzymatic pathway: CAR-mediated reduction of gallic acid to gallic aldehyde, followed by its further reduction to gallic alcohol by endogenous aromatic aldehyde reductase(s).

### Enhanced Production of Gallic Compounds by Overexpression of *qsuB*

Microbial production of gallic acid by metabolic engineering typically utilizes a biosynthetic route that converts 3-DHS, an intermediate of the shikimate pathway, to PCA [14, 16-18, 36]. This pathway offers a more direct and efficient alternative compared to routes proceeding from chorismate *via* 4-HBA and PCA, thereby improving overall yield. In *C. glutamicum*, the conversion of 3-DHS to PCA is catalyzed by 3-DHS dehydratase encoded by *qsuB* (Fig. 1). To enhance metabolic flux toward gallic acid, *qsuB* was overexpressed in engineered MA303 and PV-*Δ0324* strains carrying different plasmid combinations, followed by evaluation of gallic compound production after 40 h of flask cultivation. Flask fermentation results revealed that *qsuB* overexpression significantly boosted gallic acid production (Table 7). In GC155 (GC150 harboring pA-*qsuB*), gallic acid concentration increased from 4.30 g/l to 4.80 g/l, representing a 35% increase in yield. In GC303 (GC300 harboring pA-*qsuB*), gallic acid levels improved from 4.82 g/l to 6.59 g/l, corresponding to a 46% increase in yield. In addition to gallic acid, *qsuB* overexpression also enhanced the production of gallic aldehyde and putative gallic alcohol. Gallic aldehyde concentration in GC309 rose from 0.55 g/l to 0.64 g/l (a 72% yield increase), while putative gallic alcohol production in GC158 increased from 89.4 AU·s to 92.6 AU·s, reflecting a 36% improvement. Collectively, these results demonstrate that *qsuB* overexpression promotes efficient conversion of 3-DHS to PCA, thereby increasing carbon flux toward gallic acid and its derivatives. This metabolic rerouting offers a more efficient and high-yielding alternative to traditional chorismate-derived pathways for gallic compound biosynthesis in *C. glutamicum*.

**Fig. 3:**
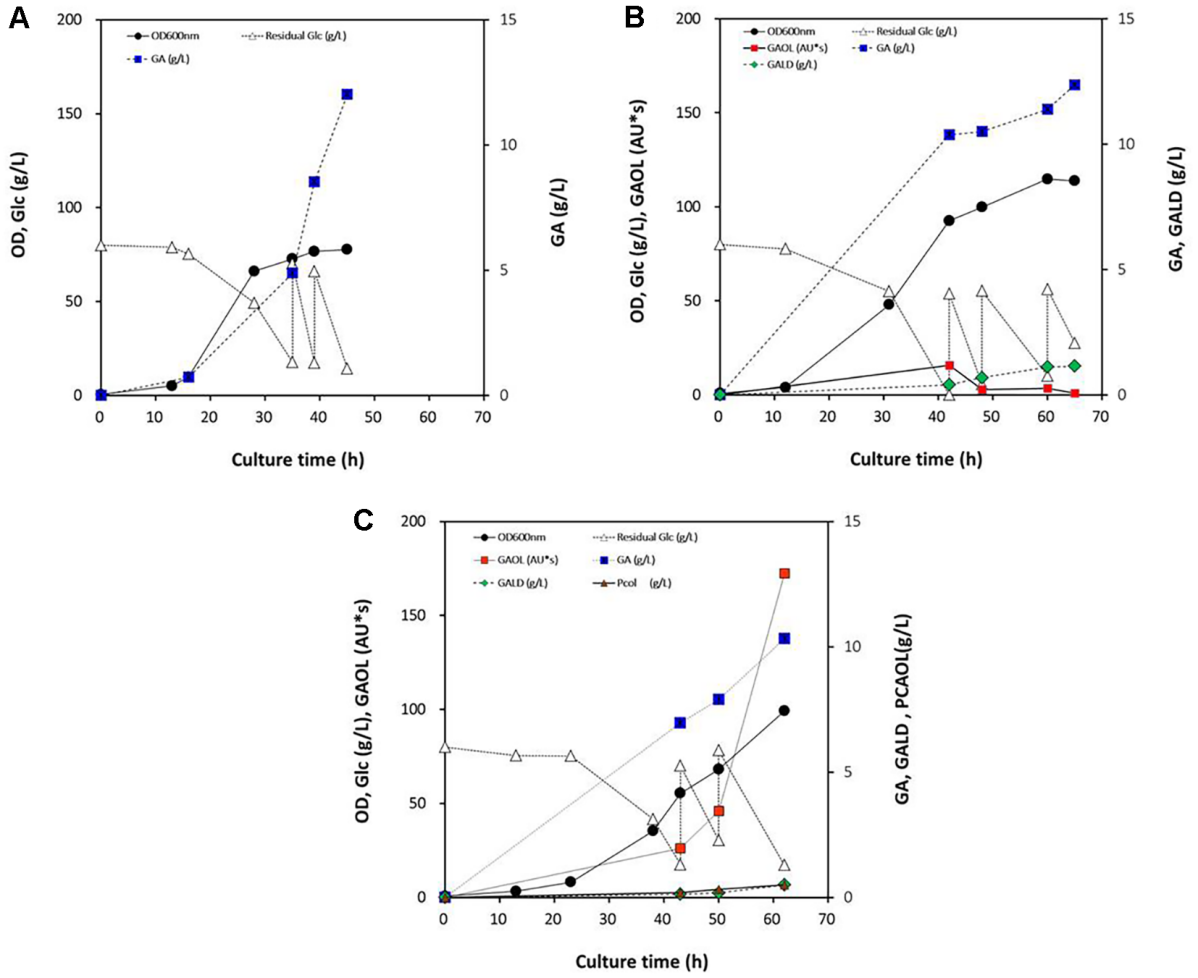
Production of gallic compounds in engineered strains GC303 (A), GC309 (B), and GC158 expressing *qusB* in a 5-l bioreactor. (**A**) Strain GC303 (PV-Δ0324 harboring plasmid pC-mFV50 and pA-QsuB); (**B**) Strain GC309 (PV-Δ0324 harboring plasmid pC-mFV50, pXT-MpCar, and pA-QsuB); (**C**) Strain GC158 (MA303 harboring plasmid pCmFV50, pXT-MpCar, and pA-QsuB). Glc, D-glucose; GA, gallic acid; GALD, gallic aldehyde; GAOL, gallic alcohol.

To further validate the production performance of engineered strains at a larger scale, fed-batch fermentation was conducted in a 5-l bioreactor using strains GC303, GC309, and GC158, which showed the highest titers of gallic acid, gallic aldehyde, and putative gallic alcohol, respectively, in flask cultures. During fermentation, GC303 (PV-*Δ0324* harboring pC-mFV50 and pA-*qsuB*) exhibited the highest gallic acid production, reaching 12.0 g/l with a yield of 8.25% after 45 h (Fig. 3A). GC309 (PV-*Δ0324* harboring pC-mFV50, pXT-MpCar, and pA-QsuB) achieved enhanced gallic aldehyde production, with a titer of 1.14 g/l and a yield of 0.76% after 65 h (Fig. 3B). Meanwhile, GC158 (MA303 harboring pC-mFV50, pXT-MpCar, and pA-*qsuB*) showed a substantial increase in putative gallic alcohol, reaching 172.4 AU*s with a semi-quantitative yield of 113.9% after 62 h (Fig. 3C). Overall, production titers of gallic acid, gallic aldehyde, and putative gallic alcohol in the 5-l bioreactor increased by 82%, 78%, and 86%, respectively, compared to flask cultures. Taken together, these results demonstrate that engineered *C. glutamicum* strains can effectively produce gallic compounds through the introduction of an artificial biosynthetic pathway and optimization of carbon flux. The successful scale-up in the 5-l bioreactor further reinforces the potential of *C. glutamicum* as a microbial platform for the industrial production of gallic compounds *via* metabolic engineering.

## Discussion

In this study, the microbial biosynthesis of gallic acid, gallic aldehyde, and gallic alcohol was achieved in *C. glutamicum* through the sequential introduction of a PobA mutant, a CAR, and modulation of endogenous AAR (Fig. 1). To initiate the biosynthetic pathway from PCA, various PobA variants from *C. glutamicum* were introduced into the PCA-producing strain MA303. Previous studies using *P. aeruginosa* PobA have shown that the Y385F mutation plays a key role in generating PCA hydroxylation activity [15]. Moreover, additional mutations such as L199V, T294A, and V349A exhibited synergistic effects when combined with Y385F, leading to increased gallic acid production *in vivo* [14, 16-18]. Consistent with these findings, the Y385F mutation from *C. glutamicum* remained essential for PCA hydroxylation. Among the additional mutations tested, L200V (corresponding to L199V in *P. aeruginosa*) exhibited a synergistic effect, whereas T294A did not contribute positively to gallic acid production (Table 3). Enzymatic assays confirmed that the Y385F/L200V double mutant had the highest PCA hydroxylation activity and was subsequently applied to the biosynthesis of other gallic derivatives (Table 5). Interestingly, the Y385F/T294A mutant derived from *C. glutamicum* did not exhibit the same enhancing effect as the corresponding mutant from *P. aeruginosa* [17]. Previous work with the *P. aeruginosa* PobA mutant indicated that conformational switching of FAD is essential for the catalytic cycle. The “out” conformation is required for flavin reduction, substrate binding, and product release, whereas the “in” conformation is necessary for substrate hydroxylation. To elucidate the structural basis for this difference, we modeled both Y385F and Y385F/T294A using AlphaFold3 and AutoDock Vina. Structural comparisons revealed a notable difference in FAD positioning:

the Y385F/T294A mutant adopted an “out” conformation of FAD due to the loss of a stabilizing hydrogen bond and alleviated steric hindrance near the isoalloxazine binding site, whereas the Y385F single mutant maintained the ‘in’ conformation (Fig. S1). A structural bias toward the “out” conformation, as seen in the Y385F/T294A mutant, may lead to reduced catalytic efficiency by promoting premature H_2_O_2_ dissociation from the flavin hydroperoxide intermediate, rather than enabling productive oxygen transfer to PCA. These results suggest that the structural and catalytic behavior of PobA from *C. glutamicum* diverges from that of *P. aeruginosa*, likely due to differences in active site architecture and cofactor interactions.

To construct a microbial biosynthetic pathway for converting gallic acid to gallic aldehyde, identification of a suitable CAR was critical. Although no CAR enzyme has been experimentally validated for gallic acid as a substrate, CARs are generally known for their broad substrate promiscuity, reducing aromatic and aliphatic acids to their corresponding aldehydes [30, 31]. Leveraging this property, we investigated microbial CARs with reported activity toward substrates structurally similar to gallic acid, including vanillic acid, PCA, 4-HBA, and benzoic acid. MpCAR has been reported to exhibit broad substrate specificity covering aromatic, heteroaromatic, and aliphatic acids and to efficiently convert cinnamic acid to cinnamaldehyde in *C. glutamicum* [33, 37]. NcCAR also displays activity toward hydroxybenzoic acids, methoxybenzoic acids, and phenylpropionic acid derivatives [35]. SrCAR is active against structurally related substrates such as vanillic acid, PCA, and 4-HBA [32]. NiCAR reduces benzoic acid derivatives and selected fatty acids and has been functionally validated in *C. glutamicum* for the conversion of 4-HB acid, PCA, and vanillic acid into their corresponding aldehydes [26, 34]. Based on these characteristics, MpCAR, NcCAR, SrCAR, and NiCAR were selected. Comparative evaluation in *C. glutamicum* revealed that MpCAR achieved the highest gallic aldehyde production, likely due to both its intrinsic catalytic activity toward gallic acid and its effective functionality in the host system (Table 6). Despite previous reports demonstrating catalytic activity of NcCAR and SrCAR toward a broad range of aromatic acids [32, 35], neither enzyme produced detectable levels of gallic aldehyde under our experimental conditions. This result suggests that NcCAR and SrCAR may exhibit either low activity toward gallic acid or limited functionality in the *C. glutamicum* host.

Our previous study showed that *C. glutamicum* possesses a strong endogenous capacity to reduce aromatic aldehydes to alcohols, primarily mediated by AAR encoded by *NCgl0324* [26]. Deletion of *NCgl0324* led to significant accumulation of 4-HB aldehyde, PC aldehyde, and vanillin, with corresponding decreases in their alcohols. In the present study, the functional role of AAR was further explored in the context of gallic aldehyde metabolism. The introduction of the PobA mutant and MpCAR into the *NCgl0324*-deleted strain PV-*Δ0324* resulted in marked gallic aldehyde accumulation, whereas the same enzymes introduced into the parental MA303 strain led primarily to the formation of putative gallic alcohol. These results strongly support the *in vivo* function of AAR in reducing gallic aldehyde to gallic alcohol. In addition, deletion of *NCgl0324* along with other aldehyde reductase genes (*dkgA* and *cg1176*) has previously been shown to enhance cinnamaldehyde production by preventing its reduction to cinnamyl alcohol [37], further highlighting the broad substrate scope of this enzyme. Collectively, AAR represents a central metabolic control point in *C. glutamicum*, governing the redox state of various aromatic aldehydes including 4-HB aldehyde, PC aldehyde, vanillin, cinnamaldehyde, and gallic aldehyde. Its disruption facilitates aldehyde accumulation by blocking their rapid reduction, while its endogenous expression promotes the production of the corresponding alcohols. From a metabolic engineering perspective, modulation of AAR provides a powerful strategy as a broadly applicable tool for fine-tuning the balance between aldehyde and alcohol synthesis in *C. glutamicum*.

Aromatic aldehydes are known to be toxic to microbial hosts, often leading to growth inhibition and metabolic burden [26, 38]. For instance, in our previous experiments with vanillin production in a 5-l bioreactor, the strong toxicity of vanillin severely impaired cell growth, resulting in the consumption of only ~50% of the initially supplied 80 g/l D-glucose and minimal biomass formation (unpublished data). In contrast, gallic aldehyde showed notably lower cytotoxicity under comparable fermentation conditions. During 5-l fed-batch fermentation, the strain GC309 successfully consumed 177 g/l of D-glucose, achieved a high cell density (OD_600_ = 114), and accumulated 1.14 g/l of gallic aldehyde after 65 h (Fig. 3B). This result suggests that gallic aldehyde exhibits considerably lower toxicity to *C. glutamicum* than other aromatic aldehydes such as vanillin, 4-HB aldehyde, and PC aldehyde [26] . One plausible explanation is that the co-production of gallic acid, which reached 12.4 g/l in the same culture, may exert a protective effect by acting as a potent antioxidant, thereby mitigating the oxidative or redox stress associated with aldehyde accumulation [1, 3]. Gallic acid is well-documented for its strong free radical scavenging activity, which could potentially alleviate cellular damage caused by gallic aldehyde or other reactive intermediates. Further investigations are warranted to clarify whether gallic aldehyde itself has inherently low toxicity, or whether its potential cytotoxicity is neutralized by the simultaneous presence of gallic acid. Systematic studies on aldehyde-specific stress responses and oxidative stress markers in *C. glutamicum* will help elucidate the cellular impact of gallic aldehyde accumulation and the possible detoxifying role of gallic acid.

To our knowledge, this work represents the first demonstration of microbial production of gallic aldehyde and gallic alcohol. Given the increasing demand for natural phenolic compounds with diverse bioactivities including antioxidant, antimicrobial, and therapeutic effects, this work provides a foundation for microbial production of gallic polyphenols. In particular, gallic alcohol, although not well characterized to date, likely possesses valuable biological functions due to its phenolic alcohol structure. The engineered *C. glutamicum* strains developed in this study offer a promising foundation for future metabolic engineering efforts aimed at sustainable biosynthesis of high-value phenolic derivatives.

## Conclusion

This study demonstrates the successful construction of a complete biosynthetic pathway for fermentative production of gallic acid, gallic aldehyde, and gallic alcohol from D-glucose in *C. glutamicum*. Expression of the PobA Y385F/L200V mutant conferred PCA hydroxylation activity for gallic acid biosynthesis, while introduction of MpCAR enabled gallic aldehyde production in an *NCgl0324*-deleted strain. In strain with intact *NCgl0324*, gallic aldehyde was further reduced to gallic alcohol by the native aldehyde reductase. Overexpression of *qsuB* enhanced flux through the shikimate pathway, increasing titers of all three compounds. Fed-batch fermentation yielded up to 12.0 g/l gallic acid, 1.14 g/l gallic aldehyde, and 172.4 AU*s gallic alcohol, confirming the potential of *C. glutamicum* as a microbial chassis for sustainable production of gallic polyphenols.

## Supplemental Materials

Supplementary data for this paper are available on-line only at http://jmb.or.kr.



## Figures and Tables

**Table 1:** Bacterial strains used in this study.

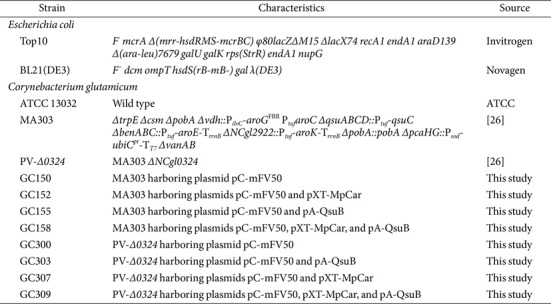

**Table 2:** Plasmid vectors used in this study.

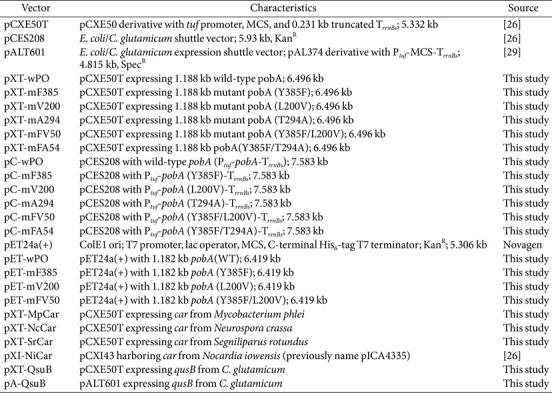

**Table 3:** Production of gallic acid and its precursors in *C. glutamicum* MA303 harboring a plasmid expressing wild-type or mutant *pobA* gene in baffled flasks.

Strain	Plasmid	OD_600nm_	Consumed glucose (g/l)	4-HBA (g/l)	PCA (g/l)	GA (g/l)	Yield (mol_4-HBA_/mol_glucose_, %)	Yield (mol_PCA_/mol_glucose_, %)	Yield (mol_gallic acid_/mol_glucose_, %)
MA303	-	50.8 ± 10.8	76.0 ± 15.3	0.82 ± 0.53	2.25 ± 1.04	< 0.05	1.40 ± 0.91	3.46 ± 1.60	-
	pC-wPO	54.1 ± 10.2	84.7 ± 13.2	0.14 ± 0.03	2.87 ± 0.52	0.56 ± 0.13	0.21 ± 0.04	3.96 ± 0.72	0.70 ± 0.16
	pC-mF385	59.2 ± 4.0	76.2 ± 14.6	0.13 ± 0.03	0.54 ± 0.24	3.88 ± 0.63	0.23 ± 0.05	0.83 ± 0.36	5.39 ± 0.87
	pC-mV200	51.6 ± 8.1	73.8 ± 16.0	0.14 ± 0.02	2.78 ± 0.98	0.26 ± 0.07	0.25 ± 0.04	4.41 ± 1.56	0.37 ± 0.10
	pC-mA294	49.5 ± 4.7	81.4 ± 9.7	< 0.05	2.52 ± 0.42	0.28 ± 0.10	-	3.62 ± 0.60	0.36 ± 0.13
	pC-mFV50	51.7 ± 5.6	71.7 ± 19.7	0.12 ± 0.02	0.07 ± 0.03	4.03 ± 0.92	0.22 ± 0.03	0.11 ± 0.05	5.95 ± 1.36
	pC-mFA54	46.1 ± 6.5	81.5 ± 12.5	< 0.05	0.16 ± 0.06	3.75 ± 0.72	-	0.23 ± 0.09	4.87 ± 0.94

Initial glucose concentration is 100 g/l. Cells were grown in 250 ml baffled flasks with 25 ml fermentation medium for 40 h.

**Table 4:** NADPH oxidation activity of wild-type and mutant PobAs with 4-hydroxybenzoate or protocatechuate as a substrate.

PobA type		NADPH oxidation activity (μmol/min/mg protein)	
		Substrate: 4-hydroxybenzoate	Substrate: protocatechuate
Wild-type PobA		2.765 ± 0.101	5.401 ± 0.205
Mutant PobA	Y385F	0.324 ± 0.022	0.681 ± 0.005
	L200V	15.87 ± 1.12	12.04 ± 0.15
	Y385F/L200V	0.512 ± 0.011	1.344 ± 0.042

**Table 5:** Product formation activity of wild-type and mutant PobAs with 4-hydroxybenzoate or protocatechuate as a substrate.

PobA type		Production formation activity (μmol/min/mg protein)	
		Substrate: 4-hydroxybenzoate	Substrate: protocatechuate
Wild-type PobA		1.742 ± 0.071	0
Mutant PobA	Y385F	0.206 ± 0.030	0.183 ± 0.015
	L200V	5.444 ± 0.734	0
	Y385F/L200V	0.166 ± 0.022	0.642 ± 0.124

**Table 6:** Production of gallic compounds in *C. glutamicum* PV-*Δ0324* and MA303 strains harboring the plasmid pC-mFV50 and a plasmid expressing *car* gene in baffled flasks.

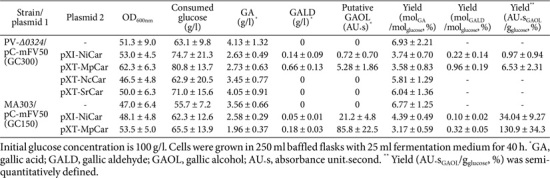

**Table 7:** Production of gallic compounds by plasmid-borne expression of qusB in engineered *C. glutamicum* MA303 and PV-*Δ0324* strains in baffled flasks.