# Supplementary material for: Metabolic Engineering of Corynebacterium glutamicum for the Fermentative Production of Gallic Compounds by Extending the Shikimate Pathway
Source: J Microbiol Biotechnol. 2025 Jun 12;35:e2504009. doi: 10.4014/jmb.2409.04009 (PMC12197807; doi:10.4014/jmb.2409.04009)

**J. Microbiol. Biotechnol. 2025. 35: e2504009**
**https://doi.org/10.4014/jmb.2409.04009**

# Supplementary Tables and Figure

## Metabolic Engineering of *Corynebacterium glutamicum* for the Fermentative Production of Gallic Compounds by Extending the Shikimate Pathway

Min-Hee Jung, Jung-Min Choi, Theavita Chatarina Mariyes, Eun-Jae Ju, and Jin-Ho Lee*

Department of Food Science and Biotechnology, BB21+, Food and Life Science Research Institute, Kyungsung University, Busan, Republic of Korea

*Corresponding author
E-mail: jhlee83@ks.ac.kr

**Table S1. Primer lists used in this study.**

| Primer | Sequence (5’ →3’) |
|---|---|
| P-F1 | gaagtccaggaggagaattcatgaaccacgtaccagtggca |
| P-R2 | cagttccctactctaagcttttatacctcgaagcgtggtagg |
| P-F3 | tccagcgctactacctgcag |
| P-R4 | gtggtaggtcgcgcccaacgaactgctctgcgaggtagcgt |
| P-F5 | acgctacctcgcagagcagttcgttgggcgcgacctaccac |
| P-R6 | ggcgccagggtggtgctagc |
| P-F7 | gctgcacacaccgtgccgccagccggagctaagggcctcaac |
| P-R8 | gttgaggcccttagctccggctggcggcacggtgtgtgcagc |
| P-F9 | ccaaaaggaagtgatctacgcaacccaccctga |
| R10 | ttgcgtagatcacttccttttgggtttttggtgct |
| P-F11 | aagaaggagatatacatatgaaccacgtaccagtggcaattat |
| P-R12 | tggtggtggtggtgctcgagtacctcgaagcgtggtaggtc |
| P-F13 | gaagtccaggaggagaattcatgcgtacatccattgccact |
| P-R14 | cagttccctactctaagcttctagtttgggattccccgct |

**Table S2. Codon-optimized version of *car* gene (*NcCar*) encoding carboxylic acid reductase from *Neurospora crassa* OR74A .**

GAAGTCCAGGAGGAGAATTC**ATG**AGCCAGCAACAAAACCCACCTTATGGTCGCCGGCTCATTCTGGATATTATC
AAGGAACGGGCATTGAATGAGCCTAATCGTGAATGGGTCTCTGTGCCACGGTCATCGGATCCGAAAGATGGTTG
GAAGATTCTTACGTACCTTGACGCATACAATGGGATTAACCGTGTCGCGCACAAACTCACTCAAGTCTGTGGTG
CAGCGGCTCCGGGATCATTCCCTACCGTGGCCTATATCGGACCTAACGATGTGCGCTATCTCGTATTTGCGCTCGG
CGCTGTCAAAGCAGGCTATAAGGCACTCTTTATCTCTACCCGTAACTCTGCAGAGGCGCAAGTAAATCTGTTCGA
ACTTACCAACTGCAACGTGCTCGTGTTTGATCAGTCATATAAGGCGACGGTGCAGCCCTGGTTGCATGAGCGCG
AAATGACGGCAATTCTCGCGCTGCCAGCAGACGAGTGGTTTCCAGCTGACCAGGAAGATTTCCCTTACAATAAA
ACGTTTGAAGAAGCTGAATGGGATCCCTTGATGGTTCTCCATACTTCTGGATCAACGGGTTTCCCGAAGCCAATC
GTCGCCCGTCAGGGAATGCTTGCGGTGGCCGACCAGTTTCATAATCTGCCTCCACGGGAGGACGGCAAATTGAT
GTGGATTGTCGAAATGTCTAAACGCGCGAAGCGGTTGATGCACCCAATGCCGCTCTTTCATGCGGCAGGAATGT
ACATTAGCATGCTCATGATCCACTATTGGGATACGCCAGGAGCTCTCGGCATCGGCGAGCGGCCACTGTCCTCGG
ACCTCGTACTTGATTATATTGAGTACGCGGACGTAGAAGGCATGATCCTTCCTCCGGCGATCCTTGAGGAACTCT
CACGGGACGAAAAGGCTATTCAGTCTCTCCAGAAGCTCAACTTCGTATCCTTTGGAGGTGGTAATCTGGCCCCG
GAAGCCGGTGATCGTCTGGTTGAGAACAACGTGACTCTTTGCAACCTGATTTCGGCCACCGAGTTCACGCCATT
CCCATTTTATTGGCAGTATGACCAGAAACTGTGGCGCTACTTTAACTTTGATACCGATCTCTTCGGTATTGACTGG
CGGTTGCATGACGGAGAGTCGACCTACGAACAGGTTATCGTGCGTAAGGATAAGCATCCGGGACTCCAGGGCTT
CTTTTACACGTTTCCCGACTCGTCTGAATACTCCACCAAAGACCTCTATAAGCGCCACCCCACCCATGAGGACTT
TTGGATCTATCAAGGTCGTGCCGATAACATTATCGTGTTCTCTAATGGTGAAAAACTGAACCCTATTACCATTGAG
GAGACCTTGCAGGGTCATCCTAAGGTAATGGGTGCAGTCGTTGTCGGTACGAATCGGTTTCAACCGGCGCTTATT
ATTGAGCCGGTTGAGCACCCAGAAACGGAGGAAGGACGGAAAGCATTGCTGGATGAAATCTGGCCCACCGTAG
TTCGTGTGAACAAAGAAACCGTCGCACATGGTCAAATTGGTCGTCAGTACATGGCCTTGTCGACGCCAGGAAA
ACCCTTTCTGCGTGCCGGCAAAGGAACGGTACTGCGTCCGGGTACGATTAACATGTATAAGGCTGAAATTGACA
AAATTTACGAAGATGCGGAGAAGGGTGTTGCGACCGACGAGGTTCCGAAACTGGATCTCAGCTCCTCAGATGC
CCTGATTGTCTCTATCGAAAAACTGTTCGAGACGTCACTTAACGCACCAAAACTGGAAGCTGACACGGATTTTT
TCACTGCAGGCGTGGATTCGATGCAGGTTATCACGGCCTCCCGGCTCATCCGCGCCGGCTTGGCAGCAGCCGGA
GTCAATATCGAGGCGTCCGCATTGGCTACCCGGGTGATTTATGGAAATCCAACTCCCAAACGCCTTGCTGATTAT
CTGCTCTCCATTGTGAACAAGGATTCCAATCAAGGAACCCTGGATAACGAACACCACGTAATGGAGGCACTGGT
CGAGAAGTACACGCGTGACCTCCCCACCCCGAAACAAAATAAGCCAGCCCCCGCTGATGAAGGACAAGTCGTT
GTAATCACTGGGACAACGGGTGGTATCGGTTCTTATCTGATTGACATCTGCTCGTCCAGCTCGCGCGTGTCGAAA
ATCATCTGCCTGAATCGTTCGGAAGACGGCAAGGCTCGCCAAACTGCTTCTTCGTCTGGACGTGGTCTGTCGAC
GGACTTCTCGAAATGTGAATTTTACCACGCAGATATGTCTCGCGCGGATCTCGGTCTTGGCCCCGAAGTCTATTC
TCGTCTCTTGTCGGAGGTTGATCGTGTCATTCACAATCAGTGGCCGGTCAATTTTAATATTGCAGTAGAGAGCTT
TGAGCCACACATTCGGGGATGTCGCAATCTGGTGGATTTTTCGTACAAGGCCGATAAGAACGTTCCAATCGTGTT
TGTGTCTTCAATTGGCACTGTAGATCGTTGGCACGACGAGGATCGGATTGTTCCCGAGGCTTCTCTGGACGATTT
GAGCTTGGCAGCGGGCGGATATGGCCAGTCAAAATTGGTTAGCAGCCTGATTTTCGATAAAGCTGCGGAGGTCT
CAGGAGTCCCGACGGAAGTAGTCCGGGTAGGTCAAGTTGCTGGTCCCTCGAGCGAGAAAGGCTATTGGAACAA
GCAGGAGTGGCTTCCAAGCATTGTGGCGTCTTCCGCTTACCTTGGTGTACTCCCAGACTCGTTGGGTCAGATGA
CTACCATTGATTGGACCCCCATTGAAGCAATCGCGAAACTTCTTCTCGAAGTTTCGGGCGTGATCGACAACGTG
CCCTTGGACAAGATCAATGGATACTTCCACGGCGTAAACCCAGAGCGTACTTCTTGGTCTGCCCTGGCCCCGGC
TGTTCAGGAATACTATGGCGATCGGATCCAGAAGATCGTTCCGCTTGATGAGTGGCTCGAGGCCTTGGAAAAGT
CACAAGAAAAAGCGGAAGACGTGACGCGGAATCCAGGTATTAAACTGATTGATACGTATCGTACCTGGTCAGA
AGGATATAAGAAGGGCACGAAATTTGTGCCGCTTGATATGACGCGGACTAAGGAATATTCCAAAACGATGCGGG
AAATGCACGCGGTCACGCCTGAACTCATGAAAAACTGGTGTCGGCAATGGAACTTC**TAA**AAGCTTAGAGTAGG
GAACTG

Underlined ATG means ORF (open reading frame) start codon; underlined TAA means ORF stop codon.

**Table S3. Codon-optimized version of *car* gene (*MpCar*) encoding carboxylic acid reductase from *Mycobacterium phlei*.**

GAAGTCCAGGAGGAGAATTC**ATG**GCATCGGAGTCGCGCGACGTACGGCTCCAACGGCGTATTGCGGAACTTTA
TGACACGGACCCTCAATTTGCTGCGGCACGTCCCGATGAAGCAGTGGCTCGTGCCGTTAATGCGCCTGGCCTGA
CTCTCTCTCAAGTTATTCGGACCGTCTTGGATAACTATGCTGATCGCCCGGCATTGGGTTACCGCGCGGTTGAGT
TTGCAGCTGATCCCGCAAGCGCTCGGACGGTCGCTCGTCTGCTTCCGCGTTTCGATACGATTACGTACCGCGAG
CTCGGAGAGCGGATCGATGCTACGACCGCCGCCCTCGGACACGACGGATTGCGGCCTGGAGAGCGCGTAGCAA
TTCTTGGCTTTTCGTCAGTGGATTATACGACTATCGACATTGCCGCGTTCAATCTGGGTGCAGTGAGCGTACCGC
TGCAGACGAGCGCTCCCCCATCGCAGCTGCGTCCCATGACGGCCGAGACTGAACCAGCCGTCATTGCAGCTTC
AGTAGATTTTCTCGATGACGCACTCGAACTTATCCGCACTGGTCATGCTCCGCGTCGTCTGGTAGTCTTTGACTT
CCACCCGGAAATCGATGATCACCGTGACGCCTTGGCTGCCGCAACTACTGGTCTTGCAGATACCGCAATGACTG
TCGAGACTCTTGATTCACTCCTCACCCGCGGTCGCACGCTCCCCGCTCCTACGGGTTATCGGGATCGGGATGATG
ACCTGGCGCTTCTCATTTACACGTCGGGATCTACGGGTGCACCAAAGGGTGCAATGTATCAGCGTCGCATGGTA
ACGAACATGTGGCGTCGCGCGACCACTGCTATTTGGGGAGGAAAGGAAGCTGCGCCGTGGCTTACCCTTAACT
TCATGCCCATGTCACATGTGATGGGCCGGGGAATCCTGAGCACTACTCTCTGTTCAGGAGGTACGGCGTATTTTG
CTGCACGGTCAGACCTGTCAACGTTGCTTGAAGACCTCGCTTTGGTCCGTCCTACGCAATTGACCTTTGTTCCA
CGCATTTGGGAGATGATTTTCCAGGAGTACCAACGTGATGTCGCCCGCCGTCCAGAGGCAGAGGTGCTGGCGGA
TCTGCGTGAACATCGGCTGGGCGGCCGTTTTCTCGCTGCGATGACGGGATCTGCGCCAATGTCGCCTGAAATGA
CGGCTTTCGCGGAATCTGTGCTTGACCTCCATCTCGTCGACGGTTACGGTTCTACGGAGGCTGGATCAATTGTGC
TTGACGGCCAGGTCCTTCGGCCTCCCGTACTTGATTACAAACTGGTTGACGTTCCTGAACTCGGCTATTTCTCCA
CGGACCGTCCTTATCCACGGGGCGAGTTGCTTGTAAAAACCGAGCTGATGTTTCCCGGATACTACAAGCGTCCA
GACATCACCGCTGAGGTGTTTGACGAGGATGGATACTATCGCACGGGAGACATCGTGGCTGAGTTGGGTCCGGA
TCGTTTGGCGTACGTGGACCGCCGGAACAACGTACTTAAACTCTCACAAGGAGAGTTTGTAACGGTGAGCAAG
CTGGAAGCCGCTTTCGCTGCGAGCCCGCTGGTGCGTCAGATTTATATTTATGGCAATTCCGCGCACCCCTACCTC
CTTGCCGTGGTAGTTCCTACCGAAGACGCACTTACCCGGTATGACGCAGCTACGTTGAAGACCGCTATCTCGGA
ATCCTTGCAGGATGTCGGACGTGCCGCGGGTTTGCAATCATACGAGATTCCACGTGACTTCCTCGTGGAGACGA
CGCCTTTCACCTTGGAAAACGGTCTCTTGACTGGTTTGCGGAAGCTCGCGCGTCCGAAGCTGAAGGAGCATTAT
GGCGAACGGCTTGAGCAATTGTACACTGAGTTGGCTGACGGTCAAGCAGAAGAATTGAAAGAACTGCGCACCC
ACGGTGCCCAGCAGCCCACTCTTACTACCGTTTCCCGTGCGGCGACCGCTCTCCTCGGCACTGCGTCTGCAGAA
GTACGGCCAGACGCACATTTCACGGACCTCGGCGGAGATTCGCTTTCCGCATTGACTTTTGGTAATCTCCTTGGT
GAAATCTATGCGGTAGAGGTGCCGGTCGGTGTGATCGTTAGCCCTGCCAACGACCTTGCCGCAATCGCTGACTA
CATCGACACGGCACGTCGCCCTGGTGATGGACGTCCTACGTTTGCCGGCGTCCACGGTGACGACGCCGCTGAA
GTCCACGCACGGGACCTCACCCTTGATCGGTTTCTTGATGATGCGACTCTTGCGGCTGCCACGGCACTCCCAGG
ACCGGCGCCCGAAATCCGTACTGTCTTGTTGACCGGCGCCACGGGCTTTCTGGGTCGCTATCTGGCGTTGGAAT
GGTTGGAGCGCATGGCAATGGTAGGAGGAACCCTCATCTGTTTGGTGCGCGGGCGGGACGACGCTGCAGCACG
CGCGCGGTTGGATCAGATTTTTGATTCGGGAGATCCCGAACTCCTTCGGCACTACCGGGAGCTTGCTGATCGTC
ACTTGGAGGTTATCGCAGGAGACAAATCCGATGCTGATCTCGGTTTGGACCGCCGCACTTGGCAGCGGTTGGCC
GACACTGTGGATCTTATTGTCGATCCTGCTGCTCTGGTAAATCATGTGCTCCCTTACCGTGAACTTTTTGGTCCCA
ATGTCGTGGGAACGGCGGAGTTGATCCGTTTGGCCCTTACGGGTCGTTTGAAGCCATACCTTTATACTTCAACTA
TCGCGGTTGGTGCAGGCATCGCTCCAGGACAGTTCACTGAAGATGCTGATATTCGCCAAATCTCGGCTACTCGG
ACTCTTGACGATTCTTACGCGAATGGTTATGCAACCAGCAAATGGGCTGGCGAAGTGCTCCTGCGTGAGGCGCA
TGATCTTTGCGGACTGCCTGTCGCAGTCTTTCGTTGTGATATGATCCTGGCGGATACTTCTTACGCGGGCCAACT
CAACGTCCCAGATATGTTTACGCGGCTCATCCTTTCATTGGTCGCCACTGGCATCGCGCCCCTTTCCTTTTATGAA
CTCGATGCAGCTGGTCACCGTCAGCGCGCCCATTACGATGGATTGCCGGTAGAGTTTGTAGCTGAAGCGGTGTC
AGCATTGGGCTTGGACGTGGCCGAAGACGGCGGTTTTGCTACGTATCATGTCATGAATCCGTACGACGATGGAA
TCGGACTCGATGAGTTTGTCGACTGGTTGACTGGTGCTGGTTACCCGATCGAGCACGTCAACGACTACGGAACG
TGGTTCCAACGCTTTGAAACTGCTATCCGGGGCTTGCCGGAACGGCAACGGCAAGCCAGCCTCCTTCCCTTGCT
GCACAGCTACCAACGTCCTCAGCCGCCAATCCGGGGAAGCGCCGCCCCAACCGATCGGTTCCGGTCAGCTGTC
CAGGATGCGAAAATCGGTCCTGATAAAGACATTCCGCATATCACTCCTGAGGTCATCGTCAAGTACGTTACCGAT
CTCCGCCTCCTGGGTCTCCTT**TAA**AAGCTTAGAGTAGGGAACTG

Underlined ATG means ORF (open reading frame) start codon; underlined TAA means ORF stop codon.

**Table S4. Codon-optimized version of *car* gene (*SgCar*) encoding carboxylic acid reductase from *Segniliparus rotundus* DSM 44985.**

GAAGTCCAGGAGGAGAATTC**ATG**ACTCAGAGCCACACTCAAGGACCCCAAGCATCTGCAGCCCATTCTCGCCT
CGCTCGGCGGGCAGCCGAGTTGCTTGCGACTGACCCCCAAGCTGCGGCTACATTGCCGGATCCGGAGGTGGTA
CGGCAAGCAACCCGTCCAGGCCTTCGTCTGGCAGAACGGGTAGACGCGATCCTTTCGGGATATGCCGACCGCC
CTGCGCTGGGACAACGGTCATTTCAAACCGTGAAGGACCCGATCACTGGTCGCTCATCAGTAGAGCTCCTCCCC
ACGTTTGACACGATCACCTACCGGGAGTTGCGTGAACGCGCGACGGCTATCGCTTCGGACTTGGCTCATCACCC
ACAGGCTCCAGCGAAGCCGGGTGACTTTTTGGCGTCAATCGGTTTTATTTCCGTTGACTATGTTGCAATCGACAT
CGCCGGTGTCTTTGCGGGTCTGACTGCCGTTCCGCTGCAAACGGGCGCAACCCTCGCAACGCTTACCGCCATCA
CGGCCGAGACCGCGCCGACCCTGTTCGCGGCCTCTATCGAACATCTCCCCACCGCCGTGGATGCCGTCCTTGCC
ACTCCTTCGGTCCGGCGTCTCCTTGTCTTTGATTATCGGGCCGGATCTGACGAGGATCGCGAGGCAGTAGAAGC
CGCAAAACGGAAAATTGCAGATGCGGGTTCTTCTGTCTTGGTCGATGTCCTTGACGAGGTAATTGCGCGTGGAA
AATCAGCGCCAAAGGCGCCGCTCCCGCCGGCGACGGACGCGGGTGATGACTCACTCAGCCTTCTGATTTATACG
TCGGGCAGCACCGGTACGCCAAAGGGCGCAATGTACCCTGAGCGGAATGTAGCGCACTTCTGGGGAGGAGTGT
GGGCAGCGGCCTTTGATGAGGACGCAGCTCCTCCGGTGCCAGCGATTAATATTACTTTTCTCCCACTTTCTCATG
TAGCCTCCCGTCTCAGCCTCATGCCTACCCTTGCACGTGGCGGACTCATGCACTTTGTGGCGAAGTCAGACCTTT
CAACTCTGTTTGAGGATCTCAAACTTGCACGTCCTACTAATTTGTTCCTCGTACCCCGTGTTGTGGAGATGCTCT
ACCAACATTACCAATCTGAGCTCGACCGGCGCGGCGTCCAAGATGGCACTCGGGAGGCTGAAGCGGTTAAAGA
CGACCTTCGGACTGGCCTCCTTGGTGGTCGTATCTTGACTGCGGGATTTGGCTCAGCGCCCTTGTCCGCAGAGC
TCGCAGGCTTTATCGAATCTTTGCTTCAGATCCATCTGGTAGACGGCTACGGATCAACCGAGGCAGGACCCGTG
TGGCGGGATGGATACTTGGTTAAACCCCCAGTAACCGACTACAAGCTGATCGATGTGCCGGAACTTGGCTATTTT
AGCACCGATTCGCCTCACCCACGTGGAGAGCTCGCTATCAAAACCCAAACCATCCTTCCCGGCTATTATAAGCG
GCCAGAGACGACCGCAGAAGTTTTCGATGAGGACGGCTTTTATCTGACGGGAGATGTTGTGGCGCAGATTGGA
CCCGAGCAGTTTGCGTACGTAGACCGCCGCAAGAACGTCCTGAAGCTCTCACAGGGAGAATTTGTTACTCTTGC
CAAGCTCGAGGCAGCTTATTCGTCTTCGCCTCTGGTTCGCCAGCTTTTTGTGTACGGAAGCTCGGAACGGAGCT
ACCTCTTGGCTGTTATCGTCCCGACGCCCGACGCACTGAAAAAATTCGGCGTTGGTGAGGCTGCGAAGGCGGC
TCTCGGTGAATCACTGCAAAAAATTGCACGGGACGAAGGTTTGCAGTCGTATGAGGTGCCTCGTGACTTTATCA
TTGAAACCGACCCCTTCACTGTAGAAAACGGCCTGCTGTCGGACGCACGTAAGTCCCTTCGGCCAAAATTGAA
GGAGCATTACGGCGAGCGTCTTGAGGCGATGTATAAAGAGCTTGCGGACGGCCAAGCCAATGAATTGCGTGAC
ATCCGGCGCGGTGTGCAACAACGGCCAACGTTGGAGACCGTTCGTCGTGCCGCTGCAGCGATGCTTGGAGCCT
CCGCAGCCGAAATCAAGCCGGATGCACACTTCACTGATCTGGGAGGCGACTCCCTCTCAGCGCTCACCTTCAGC
AACTTTCTGCATGACCTTTTCGAAGTTGATGTACCAGTCGGAGTAATCGTAAGCGCGGCCAATACGCTCGGTTCG
GTCGCAGAACACATTGACGCGCAGCTTGCGGGTGGTCGTGCTCGGCCGACTTTTGCCACGGTGCACGGCAAAG
GTTCGACGACCATTAAGGCAAGCGATCTTACCCTCGATAAGTTTATTGACGAGCAAACGCTCGAGGCTGCAAAG
CATCTTCCAAAACCCGCGGATCCCCCGCGTACGGTGCTTCTCACCGGTGCAAACGGATGGCTCGGTCGCTTCCT
GGCGCTCGAGTGGCTCGAACGTTTGGCACCCGCGGGCGGTAAACTTATCACTATTGTACGGGGTAAGGATGCTG
CACAGGCTAAGGCGCGGCTCGATGCGGCGTATGAGTCAGGTGATCCTAAGCTGGCCGGTCACTATCAGGACCTG
GCAGCCACGACTCTCGAAGTTTTGGCAGGCGACTTTTCCGAGCCACGTCTCGGCTTGGACGAAGCTACCTGGA
ACCGTCTCGCCGACGAAGTTGACTTTATCTCGCACCCTGGCGCCCTCGTCAATCACGTGCTCCCGTACAACCAA
TTGTTTGGTCCGAATGTGGCCGGTGTAGCGGAAATCATTAAGCTGGCCATCACCACGCGGATTAAGCCTGTCAC
TTACCTCTCGACCGTTGCCGTGGCAGCAGGAGTGGAACCTTCCGCTCTCGATGAGGATGGTGATATTCGTACCG
TTTCTGCCGAACGTTCGGTAGATGAAGGCTACGCCAATGGCTACGGCAATTCCAAATGGGGCGGTGAGGTACTC
CTTCGGGAGGCTCATGATCGGACGGGACTTCCTGTGCGGGTTTTCCGCTCAGACATGATTTTGGCCCATCAGAA
ATATACCGGACAGGTCAATGCCACTGATCAATTCACCCGCCTGGTACAGTCATTGCTTGCGACCGGCCTCGCTCC
TAAGTCATTCTACGAGTTGGACGCACAAGGCAATCGGCAACGTGCCCATTACGACGGAATCCCGGTTGACTTCA
CCGCAGAGTCTATCACTACGTTGGGTGGAGACGGCCTGGAAGGTTATCGTTCCTACAATGTGTTCAACCCGCAT
CGCGACGGTGTTGGCCTTGACGAGTTCGTAGATTGGCTTATTGAGGCCGGCCACCCGATCACTCGTATCGACGA
TTATGACCAGTGGCTGTCGCGGTTCGAAACCTCCCTGCGCGGCCTCCCTGAATCGAAGCGCCAAGCGTCTGTCC
TCCCACTGCTTCACGCTTTTGCCCGTCCTGGTCCCGCTGTGGATGGCTCACCCTTCCGGAATACCGTATTCCGCA
CCGATGTTCAAAAAGCTAAAATCGGAGCTGAGCATGATATCCCACACCTCGGAAAAGCCCTTGTACTGAAATAC
GCCGACGACATCAAACAGCTCGGCCTCTTG**TAA**AAGCTTAGAGTAGGGAACTG

Underlined ATG means ORF (open reading frame) start codon; underlined TAA means ORF stop codon.

## Supplementary Figure

**Fig. S1. Superimposition of Y385F and Y385F/T294A mutants.** Model structures of Y385F and Y385F/T294A are colored in orange and cyan, respectively. The isoalloxazine rings bound to each mutant are colored to match their corresponding mutant models. Docked model of substrate (protocatechuate, 3,4-dihydroxybenzoate) is colored in yellow. Hydrogen bonds are shown as blue dashed lines.

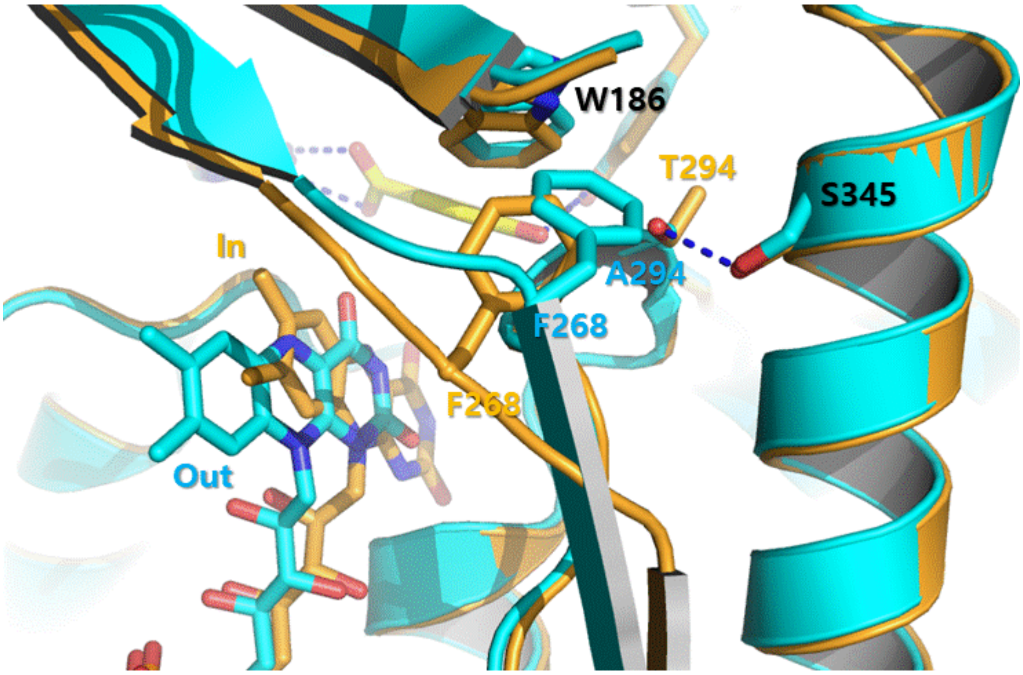

Supplement: Supplementary file 1 [file jmb-35-e2504009-supple.pdf]
